# Rise of public e-learning opportunities in the context of COVID-19 pandemic-induced curtailment of face-to-face courses, exemplified by epidural catheterization on YouTube.

**DOI:** 10.1186/s12909-023-04409-8

**Published:** 2023-06-05

**Authors:** Armin N. Flinspach, Jasmina Sterz, Vanessa Neef, Mairen H. Flinspach, Kai Zacharowski, Miriam Ruesseler, Lena Janker, Florian J. Raimann

**Affiliations:** 1 Department of Anaesthesiology, Intensive Care Medicine and Pain Therapy, University Hospital Frankfurt, Goethe University Frankfurt, Theodor-Stern Kai 7, 60590 Frankfurt/Main, Germany; 2 Institute for Medical Didactics and Clinical Simulation, medical faculty, University Hospital Frankfurt, Goethe University Frankfurt, Frankfurt, Germany; 3 Department of Trauma, Hand and Reconstructive Surgery, University Hospital Frankfurt, Goethe University Frankfurt, Frankfurt, Germany; 4 Department of Anaesthesiology, Intensive Care Medicine and Pain Therapy, Sana Clinic Offenbach GmbH, Offenbach/Main, Germany

**Keywords:** Education, Teaching, COVID-19, Instructional film and video, Obstetrics, Anesthesia, Epidural

## Abstract

**Background:**

In the context of the coronavirus pandemic, countless face-to-face events as well as medical trainings were cancelled or moved to online courses, which resulted in increased digitalization in many areas. In the context of medical education, videos provide tremendous benefit for visualizing skills before they are practised.

**Methods:**

Based on a previous investigation of video material addressing epidural catheterization available on the YouTube platform, we aimed to investigate new content produced in the context of the pandemic. Thus, a video search was conducted in May 2022.

**Results:**

We identified twelve new videos since the pandemic with a significant improvement in the new content in terms of procedural items (p = 0.03) compared to the prepandemic video content. Video content released in the course of the COVID-19 pandemic was more often created by private content creators and were significantly shorter in total runtime than those from university and medical societies (p = 0.04).

**Conclusion:**

The profound changes in the learning and teaching of health care education in relation to the pandemic are largely unclear. We reveal improved procedural quality of predominantly privately uploaded content despite a shortened runtime compared to the prepandemic period. This might indicate that technical and financial hurdles to producing instructional videos by discipline experts have decreased. In addition to the teaching difficulties caused by the pandemic, this change is likely to be due to validated manuals on how to create such content. The awareness that medical education needs to be improved has grown, so platforms offer specialized sublevels for high-quality medical videos.

**Supplementary Information:**

The online version contains supplementary material available at 10.1186/s12909-023-04409-8.

We found that new educational videos created during the pandemic were improved in terms of procedural quality even though they mostly came from private content creators. However, it remains unclear to what extent this extent this trend was maintained. Platforms containing prior review mechanisms seem promising.

## Introduction

The training of medical students is in a constant state of transformation [1]. This is due in part to the enormous increase in knowledge in medicine over the past decades [2]. As a result, the concepts of knowledge transfer are also changing [3]. In academic training and subsequent specialist training, digital media have been used for years to enhance face-to-face teaching [4]. In the context of growing digitalization, teaching concepts are increasingly being offered in hybrid form. To meet the rising demand, there are master programmes in medical education as well as professional institutes for medical didactics at medical faculties [5, 6]. In the context of medical education, videos can be of enormous help to allow students to visualize skills before first practising on a patient, to repeat partial steps at their own learning pace and to improve the learning curve [7–9]. Videos help to create an environment with a focus on the student and his or her activity [10]. The increasing role of video material in student education and its influence on students’ learning engagement have been described repeatedly [11, 12]. However, the quality of such video material varies considerably and is particularly difficult to assess in the case of material that is easily available to the public, such as on the YouTube platform [13].

The COVID-19 pandemic was a caesura in many areas of life. To prevent the spread of infections, many governments worldwide decided to impose far-reaching restrictions on freedom of assembly. In addition to restrictions on sports and recreational events, which were prominently discussed in public, corresponding regulations also applied to medical training and teaching events in attendance [14, 15]. In view of the urgent need for human resources of qualified staff, the dilemma between increased infection risk and critically necessary training became apparent. In the context of this development, online-based learning was presented as an excellent option for continuing education [16–18]. Additionally, expanding availability of more educational video material emerged [19]. Previous barriers to creating videos among academic clinicians due to the necessary expense and resources may have diminished dramatically in the context of compulsory e-learning [20, 21].

In modern anaesthesiology, neuroaxial procedures are among the established and most frequently used methods of peripartum and procedural analgesia. Discovered at the turn of the century by August Bier, this extraordinarily effective technique for reducing birth pain has become indispensable [22]. Recent studies have shown that worldwide, the majority of all women who give birth use a neuroaxial procedure as part of their delivery. However, there are still many myths and misconceptions that shape the public image [23]. Learning to place epidural catheters is one of the most important anaesthesiological skills and should therefore be taught, learned and mastered early by future specialists in this field [24, 25].

Based on a preliminary investigation of the quantity and quality of available video material on the public video platform YouTube regarding epidural catheterization, we aimed to investigate the current pandemic video material [13]. Within the previously published work, a questionnaire-based evaluation of the procedural and didactic quality of the available video material was conducted. The questionnaire used in this study was designed and tested for validity and reliability within the framework of a previous study project, resulting in an interrater reliability of 0.975. Unfortunately, among the 16 videos found in the previous investigation, only two videos showed satisfactory procedural quality (> 60% of questionnaire fulfilment), while eleven were able to achieve > 50% of the points with regard to didactic aspects. In this respect, the authors identified huge potential for improvement with regard to the prepandemic situation, which required investigation. In addition, the present study investigated the extent to which progressive algorithms on the platform side facilitate the retrieval of suitable video material.

## Methods

The study was conducted according to the ethical principles of the Helsinki Declaration (Ethical Principles for Medical Research Involving Human Subjects) [26]. Since the present study only involved an evaluation of publicly available videos and was not considered clinical research on human subjects, no ethical vote was required according to the guidelines of the ethics committee of the University Hospital of Frankfurt.

### Acquisition and evaluation of videos

To find suitable newly published video material on the YouTube Platform (https://www.youtube.com), a search was conducted analogous to the previous investigation in May 2022 in the German and English languages using the same search terms. The terms “epidural” and “peridural” were collected individually and in combination with the keywords “birth”; “obstetrics”; “delivery”; “catheter”; “anesthesia” and “anaesthesia”. Over 500 videos were examined by independent investigators regarding their suitability to demonstrate peridural anaesthesia. Twelve videos were acquired for study purposes (the last video access was on December 22nd, 2022). For each video, the uploader/producers, date of upload, and runtime were recorded for analysis purposes. In addition, the videos were categorized according to the content creators. For this purpose, a consensus was reached among the investigators to categorize the creators of the content into one of three groups: university/ medical association, clinic/ medical business provider, and private content creator.

The video material obtained was assessed by 9 independent raters according to the pre-existing checklist. The panel of raters included 3 medical students in advanced clinical training, 2 long-term experienced masters in medical education, and 4 experienced anesthesiologic specialists with extensive expertise in neuroaxial procedures. Adjusted for the prepandemic study, the new assessment was performed by the same clinical experts, with the exception of the student raters, for improved comparability, despite previously demonstrated low interrater reliability. The subsequent statistical investigation was thus based on 9 independent rater results. The subsequently applied checklist for video evaluation was bipartite and consisted of a section addressing the procedural aspects of peridural catheter placement, which consisted of 4 subsections (initial preparation, sterile handling, punction, post-procedure) with a total of 20 items ordinally scaled from 0 (inapplicable) through 1 (incorrect) to 2 (fully met). A second didactic checklist section with 4 subcategories and a total of 21 items using a five-point Likert scale completed the assessment. The differentiated questionnaire development as well as structure and validation process have already been published and can be accessed open access (10.3390/jcm11061726) [13].

### Statistics

Data were collected using Windows Excel (Office 2021, Microsoft Corporation, Redmond, WA, USA). A statistical analysis plan was designed prior to the study. Data analysis was conducted using SPSS (Ver. 29, IBM Corp., Chicago, IL, USA). Data with continuous scales are presented as mean (± standard deviation), and data with categorical scales are presented as frequencies and percentages. Interrater reliability was analyzed using a intraclass correlation coefficient. Correlations were examined with a Spearman’s rho test, as well as a Mann-Whitney U test. A p-value of < 0.05 was considered statistically significant.

## Results

**Fig. 1:**
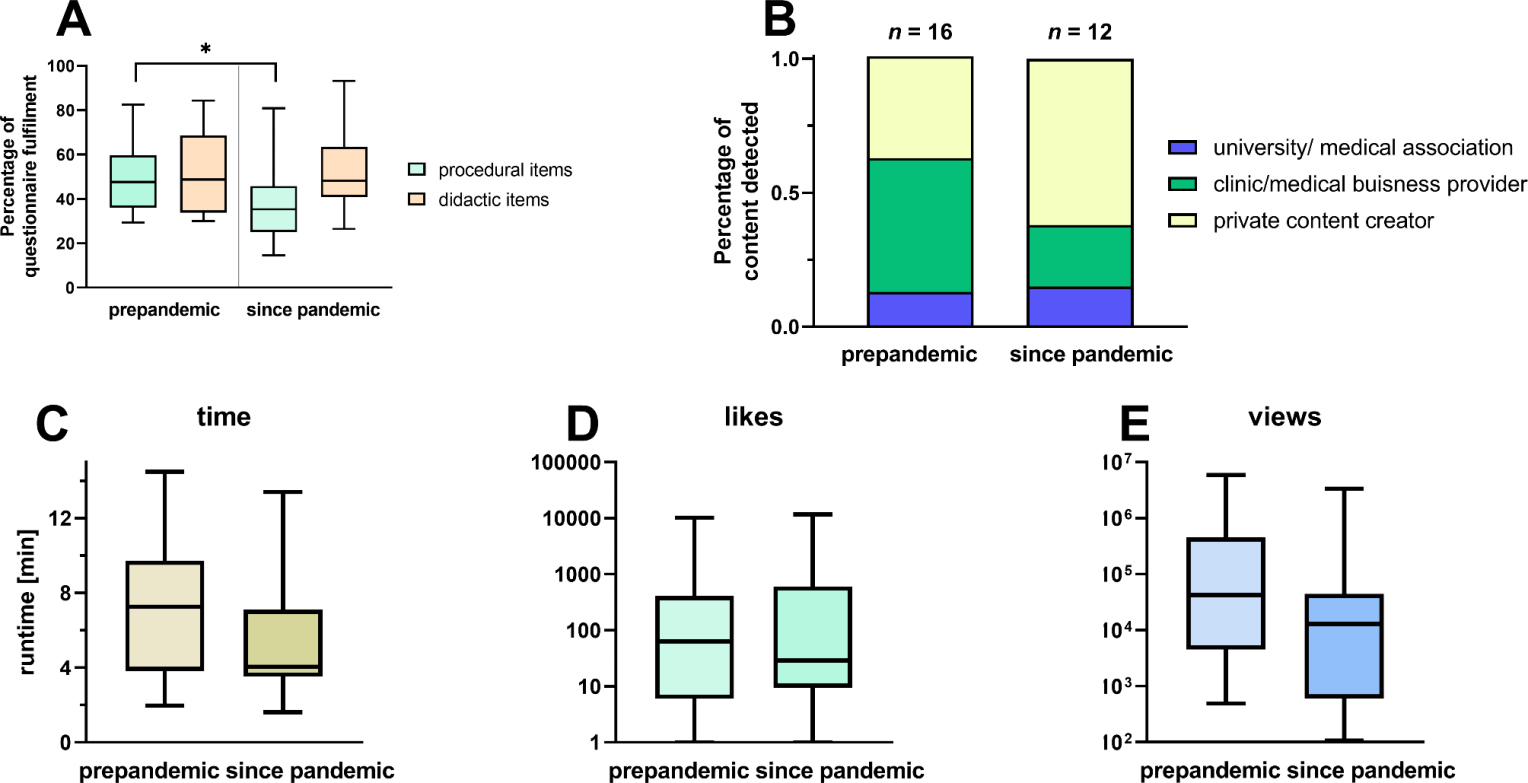
**A-E**: Comparison of acquired YouTube video material. Analysis of the pre- and post-pandemic video content on YouTube in terms of epidural catheter placement upon new research. **A**) Comparison of item fulfilment differentiating procedural and didactic items. **B**) Differentiation of content creators regarding production background. **C**) Runtime differences. **D**) Number of likes at the time of survey. **E**) View differentiation at the time of survey. * p = 0.03

We found that twelve new videos were published during the study period. The video characteristics in terms of the fulfilment of the procedural and didactic requirements for items of the established questionnaire are shown in Fig. 1A. We observed a significant improvement in the new content since the beginning of the pandemic in terms of procedural items (0.77 ± 0.34 vs. 1.01 ± 0.30; p = 0.033). Video content released during the course of the COVID-19 pandemic was more often created by private content creators (Fig. 1B). In terms of video runtime, the median duration of the videos was shortened (p = 0.227) (Fig. 1C). Taking into account all videos on epidural catheterization, a significantly longer playing time was found for videos from university and medical societies compared to private content creators (724 ± 159.5 s vs. 342 ± 153.6 s; p = 0.047). We did not observe any significant changes in the frequency of likes or views (p = 0.174; p = 0.732) (Fig. 1D-E).

The comparison of the content of the video material produced with regard to didactic aspects showed no significant improvement overall in the content produced in the context of the pandemic (p = 0.837). The analysis of the individual items showed that there was a partly significant improvement with regard to the subject-related items. Similarly, a few significant improvements as well as deteriorations in the didactic section could be found. A list of trends and significant changes in the individual items can be found in Table 1.

**Table 1:** Questionnaire completion rate of the videos detected with level of significance regarding change during pandemic

		prepandemic	since pandemic	p-value
		*n* = 16	*n* = 12	
**procedural items**		maximum 2 points		
	CTG monitoring during procedure	0.57	0.04	0.051
	hygienic hand disinfection	0.19	0.63	0.055
	wearing sterile gloves	1.09	1.16	0.058
	puncture site selection	0.74	1.12	**0.043**
	needle guidance	0.57	1.57	**> 0.001**
	loss of resistance technique	0.56	1.36	**0.005**
	catheter insertion technique	1.07	1.69	**0.009**
	catheter insertion depth	0.29	0.90	**> 0.001**
	aspiration testing (CSF, blood)	0.26	1.09	**0.001**
	postinterventional vital monitoring	0.63	0.17	0.059
**didactic items**		maximum 5 points		
	detailed process flows/work steps	2.35	2.87	0.060
	sensible order of content	3.73	3.98	**0.029**
	content related aspects of hygiene	2.28	2.67	**0.033**
	abbreviations avoided or explained	3.01	1.83	**0.011**
	referencing background information	1.26	0.98	**0.029**

Data presented as mean value of the achieved item score. With regard to the procedural items, 0–2 points could be awarded in the validated questionnaire, for the didactic items 0–5 points. Abbreviations: CTG, cardiotocography; CSF, cerebrospinal fluid

The statistical evaluation of the correlation coefficient showed no difference in the still very good interrater reliability (prepandemic p = 0.975 vs. pandemic and after p = 0.974) [27, 28].

## Discussion

We were able to identify twelve new videos regarding peridural catheterization over the course of the pandemic. Although the runtime of the videos was decreased, there was a tendency towards an improvement in quality. In addition, there was a marked increase in the amount of private content created and uploaded over the course of the pandemic. The fact that the COVID-19 pandemic profoundly changed learning and teaching worldwide is unquestioned. However, it is largely unclear what long-term changes in education will arise from this fact [29]. In the area of traditional school education among children and young adults, the need to avoid face-to-face classes due to the risk of virus transmission emerged to the same extent as in the academic environment [30]. While hybrid learning concepts already existed in many academic faculties and students were therefore well equipped with appropriate devices, this was not transferable to the area of traditional school education [31]. This situation caused numerous problems; however, digital training resources increased in nonprofessional education to an extreme extent in the context of the pandemic that were difficult to transfer to medical training. Although new video material on the placement of epidural catheters appeared during the study period, it did not appear to the extent we expected since our previous reporting [13]. Using the specific example we chose, it was possible to compare the prepandemic and the pandemic period.

By comparison, the video content we analysed in the pandemic context showed a decreased average running time, which may be attributed to the increased representation of private content creators. Thus, the increased procedural quality of the videos seems interesting. Prepandemic videos showed a significant correlation between longer runtimes and the quality of the content as well as the didactic quality. However, experts agree that a video should not be too long since the limit of expected working memory capacity is only approximately ten minutes [32]. In the recently screened material, 10 videos were below this ten-minute runtime. Although technological progress has made it much easier for private content creators to produce material due to camera requirements for high-quality audio and video recording, the subsequent editing and the implementation of additional content, there is no significant difference in the area of didactic quality. This may explain the primary improvement in the procedural section as the hurdle for physicians and didactic specialists to create educational videos has been significantly lowered.

We were able to demonstrate a significant improvement in the procedural content quality of the newly created video material that could not be shown for the didactic questionnaire section. While significant improvements or trends could be found in the majority of items, a deterioration was revealed with regard to the procedural item of “postinterventional vital monitoring” (p = 0.059) and the didactic items “abbreviations avoided or explained” (p = 0.011) and “referencing background information” (p = 0.029). The cause of this deterioration remains largely unclear. The focus of the predominantly private content creators may be more towards their followers and thus a specialized audience, so the increasing use of medical terms and abbreviations could be explained. In this case, suitable referencing would also be appropriate; however, this also significantly decreased. Considering only the improved procedural content quality, it appears that awareness of the need for online-based learning has grown, but training in the creation of more professional instructional videos is still lagging. There are now several validated manuals for creating high-quality instructional video material as well as corresponding public resources (e.g., National Institutes of Health (NIH)) [33]. Not to be left unmentioned in this context is the significant improvement in individual didactic items such as “sensitive order of content” and “content-related aspects of hygiene”, even though there was no significant improvement in the didactic section of the questionnaire overall.

It is becoming increasingly apparent that in addition to the quantity of available information and video material, the quality of both content and didactic aspects is of increasing importance. In this regard, in addition to the questionnaires we used, the open educational resource movement has determined important points of presentation, especially for public video material [34]. Regarding the COVID-19 pandemic, the quality of information in relation to the growing number of disinformation and conspiracy theories around the coronavirus and the respective vaccines has been brought into focus at an extreme level, particularly for the medical community. Beyond the field of professional medical journalism, there have been claims that major platforms such as Twitter, Facebook, YouTube and Instagram should quickly block medical disinformation from being disseminated, while questions have been raised about how to address the alleged publication of professional information on preprint servers without a peer-review process.

The awareness that the visibility of professional medical training material in terms of quality and reliability needs to be improved has grown beyond the world of medical educational experts. One of the most exciting projects is the decision of the Alphabet consortium to expand the YouTube platform to add YouTube Health (https://health.youtube/), which aims to provide a platform on which high-quality medical knowledge can be shared via video. Under the claim “We are working on ways to expand eligibility to our health features on YouTube to better connect users with health sources”, the program is an attempt by the company to cooperate with large, respected organizations such as the American Heart Association, the New England Journal of Medicine, the World Health Organization (WHO) and many others [35]. It will be interesting to see how the quality of the video material available in the field of medicine on YouTube and the open, mainly unknown online sources for professional video material such as the NIH website, MEDtube or the archive of open educational resources will change in the coming years [36, 37].

The findings of our analysis are limited because the search was conducted exclusively for videos related to a single medical skill. Although epidural catheters are a common topic of video content, there are few videos on teaching catheter placement, so our study could only include twelve new videos. In addition, only material available at the time of the study could be considered, and the extent of projects currently producing new educational videos could not be taken into account.

## Conclusion

We found that within the pandemic situation, twelve new videos were uploaded on YouTube regarding epidural catheter placement. These videos were significantly improved in terms of the procedural item section when compared using a validated questionnaire, although the majority of the new videos were uploaded by private content creators. However, it remains unknown to what extent there is a trend towards increasingly privately created educational content with equally high quality. Adequate control mechanisms based on prior review processes seem to be promising with regard to specific subplatforms.

## Electronic supplementary material

Below is the link to the electronic supplementary material.

Supplementary Material 1

## Data Availability

The videos evaluated in the current study are accessible on the public platform YouTube (https://www.youtube.com) under the specified search terms (Links can be found in Supplement I). The datasets generated and/or analysed during the current study are included in this published article or not publicly available due national law but are available from the corresponding author on reasonable request.

## List of abbreviations

CTG: cardiotocography
CSF: cerebrospinal fluid
COVID-19: coronavirus disease 2019
NIH: National Institutes of Health (NIH)
WHO: world health organization
