# Supplementary material for: Rise of public e-learning opportunities in the context of COVID-19 pandemic-induced curtailment of face-to-face courses, exemplified by epidural catheterization on YouTube
Source: BMC Med Educ. 2023 Jun 5;23:406. doi: 10.1186/s12909-023-04409-8 (PMC10240447; doi:10.1186/s12909-023-04409-8)
Supplement: Supplementary file 1 — Supplementary Material 1 [file 12909_2023_4409_MOESM1_ESM.docx]

**Supplement I**

Pro Corona Video material:

https://www.youtube.com/watch?v=IoBOBZQ1Wss

https://www.youtube.com/watch?v=CGhjMYSr18M

https://www.youtube.com/watch?v=-LCTy18nzis

https://www.youtube.com/watch?v=wbti9kKcuEw

https://www.youtube.com/watch?v=2tw-SXI3wKU

https://www.youtube.com/watch?v=VBtgseSpPMc

https://www.youtube.com/watch?v=6dQ3QourwGk

https://www.youtube.com/watch?v=0HQOlgUKhPI

https://www.youtube.com/watch?v=9_y8gnZZDaQ

https://www.youtube.com/watch?v=uNDcf3Vw1vo

https://www.youtube.com/watch?v=ODpsxDGi-gU

https://www.youtube.com/watch?v=8SRQmLkIG7s

https://www.youtube.com/watch?v=QhhJOocXu0A

https://www.youtube.com/watch?v=qXbTEIV3t1o

https://www.youtube.com/watch?v=_URgMM4yTIQ

https://www.youtube.com/watch?v=6NaSOwd6jJo

Videos released in the context of the COVID-19 pandemic:

https://www.youtube.com/watch?v=UKNg7cYamRU

https://www.youtube.com/watch?v=zke71z-aers

https://www.youtube.com/watch?v=bTAa_8KZYPE

https://www.youtube.com/watch?v=ATccI4fkzU0

https://www.youtube.com/watch?v=qURQ9bjCg1g

https://www.youtube.com/watch?v=27PtQi8ydMs

https://www.youtube.com/watch?v=wkoYKpOSbVQ

https://www.youtube.com/watch?v=R7nBpTPtSkM

https://www.youtube.com/watch?v=bTAa_8KZYPE

https://www.youtube.com/watch?v=z47Ja5eVt2k

https://www.youtube.com/watch?v=DtzI5bX7NyA

https://www.youtube.com/watch?v=fBqTe3J30K0

https://www.youtube.com/watch?v=0E4BHL4wAmE

Last video access on December 22^nd^, 2022.
